# Iodized Salt in Cambodia: Trends from 2008 to 2014

**DOI:** 10.3390/nu7064189

**Published:** 2015-05-29

**Authors:** Arnaud Laillou, Borath Mam, Sam Oeurn, Chantum Chea

**Affiliations:** 1 UNICEF, Maternal Child Health and Nutrition Programme, no. 11 St. 75, 12000 Phnom Penh, Cambodia; E-Mail: sun@unicef@unicef.org; 2 National Sub-Committee for Food Fortification, Ministry of Planning, 386 Monivong Blvd., 12000 Phnom Penh, Cambodia; E-Mails: borathmam@yahoo.com (B.M.); chantumch@gmail.com (C.C.)

**Keywords:** salt, iodization, Cambodia, trends

## Abstract

Though the consequences of nutritional iodine deficiency have been known for a long time, in Cambodia its elimination has only become a priority in the last 18 years. The Royal Government of Cambodia initiated the National Sub-Committee for Control of Iodine Deficiency Disorders in 1996 to fight this problem. Using three different surveys providing information across all provinces, we examined the compliance of salt iodization in Cambodia over the last 6 years. Salt samples from the 24 provinces were collect at the household level in 2008 (*n* = 566) and 2011 (*n* = 1275) and at the market level in 2014 (*n* = 1862) and analysed through a wavelength spectrophotometer for iodine content. According to the samples collected, the median iodine content significantly dropped from 22 mg/kg (25th/75th percentile: 2/37 mg/kg) in 2011 to 0 mg/kg in 2014 (25th/75th percentile: 0/8.9 mg/kg) (*p* < 0.001). The proportion of non-iodized salt within our collected salt drastically increased from 22% in 2011 to 62% in 2014 (*p* < 0.001). Since the international organizations ceased to support the procurement of iodine, the prevalence of salt compliant with the Cambodian declined within our samples. To date, the current levels of iodine added to tested salt are unsatisfactory as 92% of those salts do not meet the government requirements (99.6% of the coarse salt and 82.4% of the fine salt). This inappropriate iodization could illustrate the lack of periodic monitoring and enforcement from government entities. Therefore, government quality inspection should be reinforced to reduce the quantity of salt not meeting the national requirement.

## 1. Introduction

Iodine deficiency disorders have long been recognized as a significant public health issue in Cambodia. In the late 1990s, a nationally represented survey showed that 17% of primary school children had a goiter (unpublished reports [1]). To fight this problem, the Royal Government of Cambodia created the National Sub-Committee for the Control of Iodine Deficiency Disorders (NSCIDD), which focuses on Universal Salt Iodization as the primary intervention strategy to improve the population’s iodine status.

Salt is one of the main condiments used in Cambodia. Based on salt production data and imports data, Cambodia uses roughly 80% of the salt for human and animal consumption, while another 20% goes for industrial purposes [2]. In addition, roughly 85% of the salt used in the country is produced in Cambodia, mostly by solar plants by the salt producers’ community of Kampot and Kep (SPCKK) [2]. The average consumption of salt per capita in Cambodia amounts to approximately 15 g/day, including use of iodized salt supplied to the processed food industry and used in fish sauce, pickles, and other processed foods [2].

Although production of iodized salt started in 1999, there had been only limited success in producing it in large quantities. According to the 2000 Cambodian Demographic Health Survey [3], the household consumption of iodized salt was only 13%. This was due to the existence of many small to medium-scale salt producers in several provinces and the lack of mandatory legislation in place [2]. Lessons were learned after several years of implementation of iodization in Cambodia, where development of a salt producers’ association called the Salt Producers Community of Kampot and Kep (SPCKK including 167 producers) and the 2003 Sub-Decree No. 69 on mandatory iodization of salt greatly increased the supply of iodized salt. Conkle *et al.* [4] showed that the number of Cambodian households using iodized salt grew from 28% in 2004 to 70% in 2011 following the introduction of several laws and regulations.

Because of salt iodization, the median urinary iodine levels of Cambodian schoolchildren remained the same between 2008 and 2011 around 236 μg/L [4]. In fact, those children with extremely excessive levels of iodine (greater than 500 μg/L) increased dramatically from 5.5% to 16.0% over the same period [2,5]. Thus, Cambodian schoolchildren are either (I) consuming excessive amounts of salt or (II) receiving iodine from sources in addition to salt. Since 2010, the United Nations Children’s Fund (UNICEF) has stopped supplying potassium iodate to the salt producers to ensure that producers are more in charge of the iodization program and guarantee long-term sustainability. The recent Cambodian National Food Security and Nutrition Strategy [6] highlighted the importance of continuing a fortification program to ensure appropriate nutrient intake and strengthen the quality control of related products. Using three different surveys providing information across all provinces, the purpose of this paper was to assess the compliance of salt iodization in Cambodia over the last 6 years. Due to its geographical position and its many borders with Vietnam and Thailand, the authors were also investigating the potential difference of iodized salt in the market between provinces.

## 2. Materials and Methods

### 2.1. Data Sources

During the 2008 and 2011 iodine nutrition surveys [2,5], three schools were randomly selected from each provinces using Random Number Table. In total, 72 schools were selected, one school was selected from an urban area and two schools from a rural area. Children from those schools provided salt available at the household. In total, salt was collected from respectively 2329 and 2310 schoolchildren aged 8 to 10 in 24 provinces. All salt were tested through a rapid test kit (For the analysis, we included three UNICEF and Ministry of Planning surveys giving data from every provinces) for the presence or not of iodine [2] and found that 31% in 2008 and 70% in 2011 of the collected salts were iodized. A sub-sample (presented in our paper) was than tested through a wavelength spectrophotometer for iodine content: 566 samples in 2008 were and 1275 in 2011.

For the analysis, we included three UNICEF and Ministry of Planning surveys giving data from every province.

In 2014, a team from the Ministry of Planning travelled to every province in the country and selected all salt marketed at the local markets. Those markets were selected randomly within a list of known markets in urban and rural settings. In total, 1862 samples of salt were collected in 24 provinces and tested with the WYD for iodine content.

### 2.2. Iodine Measurement

All salt samples were identified as “coarse” or “fine”. According to Cambodian standards, coarse salt is produced through solar evaporation, a process highly dependent on good weather conditions. Then once the sea water evaporates, big crystals of a diameter between 0.5–1 mm are formed and this what is defined as coarse salt. It is either sold as-is or goes through refineries where it is boiled into shallow-well reservoirs to a finer grain. Coarse salt is iodized with potassium iodate by spraying methodology while fine salt by a dry mixing method.

The same methodology and equipment were used for the three surveys. A small amount of salt (about 10 grams in a zip locked bag) was taken from each salt bag and the quantity of iodine was determined by a WYD tester. The WYD iodine checker (Kejing, Tianjin, China with (within the range of 5 to 90 mg/kg)) is a simple single wavelength spectrophotometer with the function of LCD readout. The WYD iodine checker measures the iodine level (mg/kg) in salt based on the absorption of the iodine-starch blue compound at 585 nm [7]. To perform the analysis, the following steps need to be implemented: weigh 1.0 gram of iodized salt in 50 mL tube, and add 10 mL of distilled water, 2 mL of KI-starch solution and 2 mL of H_3_PO_4_, shaking the tube until the salt was dissolved in the solution thoroughly, then add distilled water to make 50 mL and shake the tube evenly. Insert the cell which contains this solution in the cell holder of the WYD, the concentration is directly read out on LCD.

To ensure the quality of the measurement, the device was calibrated every 50 analysis or before any new measurement starts. Each time two calibrations are made by using (I) distilled water for the zero point; and (II) standard solution at 50 mg/kg provided by the manufacturer. In addition, 10% of the samples were duplicated and a variation of less than 5% was found.

### 2.3. Statistical Analysis

Data management, including quality checks, and analysis was performed with SAS^®^ 9.2 software (SAS, V9.2; SAS Institute, Cary, NC, USA). Each sample was also categorized into several groups: (I) no iodine content (0 mg/kg); (II) low content (0.1–14.9 mg/kg); (III) medium low content (15–29.9 mg/kg); (IV) meets government requirement (30–59.9 mg/kg) and (V) excessive (above 60 mg/kg). Descriptive statistics were used to examine iodine concentrations over the years. Iodine Concentration distribution curves (2008, 2010 and 2014) were asymmetric according to the test of Kolmogorov-Smirnov and therefore only non-parametric tests were performed. Median and interquartile range values of the iodine concentrations are presented and disaggregated by year and type of salt (coarse/fine salt). Iodine Concentration (continuous variables) was compared by groups by using the non-parametric Mann-Whitney U-test (two groups) for independent variables. Associations between prevalence of the different categories of iodization (from no iodine to excessive) and years were assessed using univariate logistic regression models (surveylogistic procedure). Results were considered significant at *p* < 0.05.

## 3. Results

**Figure 1:**
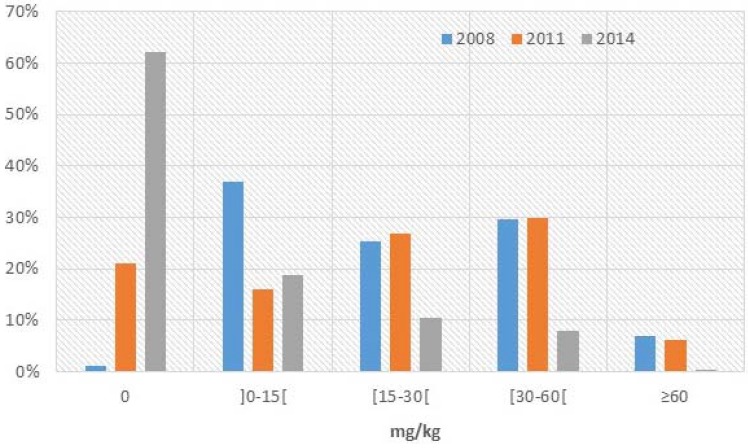
Percentage of salt in each iodine content category for all salt according to WYD testing.

First considering all salt, the median iodine level did not change between 2008 and 2011 (*p* > 0.05). The iodine levels were respectively 18.0 mg/kg (25th/75th percentile: 2.0/39.0 mg/kg) in 2008 and 22.0 mg/kg (25/75th percentile: 2.0/37.0 mg/kg) in 2011. In 2014, the median iodine content dropped significantly comparing to both year 2011 and 2008 to 0.0 mg/kg (25th/75th percentile: 0.0/8.9 mg/kg) (*p* < 0.001). In addition, the proportion of all salt without any iodine (Figure 1) increased from 1.3% in 2008 to 21% in 2011 (*p* < 0.001) and finally to 62.2% in 2014 (*p* < 0.001). The proportion of salt meeting government requirements was stable between 2008 and 2011 towards 30% (*p* > 0.05) but then decreased significantly to 8% in 2014 (*p* < 0.001). The proportion of salt with a concentration between 15 and 30 mg/kg followed the same trend as the “meeting government requirements” group. It increased but not statistically significantly from 25.4% in 2008 to 26.8% in 2011 (*p* > 0.05) then decreased to 10.4% in 2014 (*p* < 0.001). The supplementary figure shows the 2014 iodine values (mg/kg) in all salt sorted by lowest to highest iodine value.

Data on coarse salt is even more problematic (Table 1). The median iodine level of coarse salt increased significantly (*p* < 0.01) between 2008 and 2011 as it went respectively from 13.5 mg/kg (25th/75th percentile: 1.0/30.25 mg/kg) to 16.0 mg/kg (25th/75th percentile: 0.0/30.0 mg/kg). The median iodine content in 2014 dropped significantly comparing to year 2011 and 2008 to 0.0 mg/kg (25th/75th percentile: 0.0/0.0 mg/kg) (*p* < 0.001). Data on fine salt are a bit different, as 22.1% of the fine salt was adequately iodized compared to 0.7% of coarse salt. Differently from the coarse salt, the median iodine level of fine salt did not change to stay towards 30.0 mg/kg between 2008 and 2011 (25th/75th percentile respectively 16.0/48.0 mg/kg in 2008 and 14.5/40.0 mg/kg in 2011) (*p* > 0.05) and then significantly dropped to 3.6 mg/kg (25th/75th percentile: 0.0/16.5 mg/kg) in 2014 (*p* < 0.001).

**Table 1:** Iodine content of coarse and fine salt based on WYD testing over seven years.

	Year	*N*	No Iodine	Low Iodine	Low-Medium Iodine	Meets the Gonernment Guidelines	Excessive
			0 mg/kg	0.1–14.9 mg/kg	15–29.9 mg/kg	30–59.9 mg/kg	≥60 mg/kg
Coarse Salt	2008	314	1.9%	50.0%	22.0%	20.7%	5.4%
	2011	634	33.1%	15.8%	25.2%	21.8%	4.1%
	2012	314	93.7%	5.7%	0.4%	0.3%	0.0%
Fine Salt	2008	634	0.4%	20.2%	29.8%	40.9%	8.7%
	2011	742	9.0%	16.4%	28.2%	38.1%	8.3%
	2012	242	41.4%	27.7%	17.1%	13.1%	0.7%

Between 2008 and 2011 (Table 1), the prevalence of non-iodized coarse and fine salt increased significantly (*p* < 0.001) while the prevalence of low iodized coarse salt decreased significantly over the same period (*p* < 0.001). All other categories of salt remained statistically the same. In 2014, the iodine contents of all the sub-categories presented in Table 1 were different compared to 2011 (*p* < 0.001).

**Figure 2:**
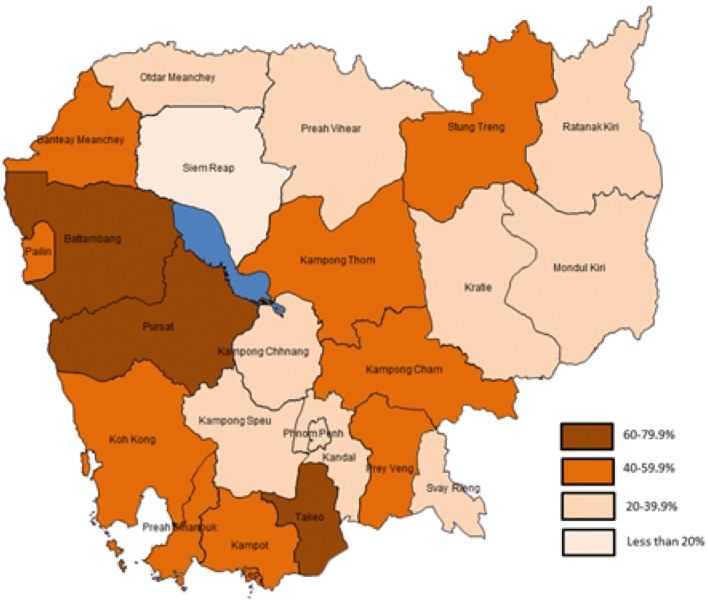
2014 Proportion (in %) of non-iodized fine salt in different provinces of Cambodia.

According to the data collected in 2014, the prevalence of non-iodized coarse or fine salt can be significantly different between provinces (*p* < 0.001). The map below (Figure 2) shows the prevalence of non-iodized fine salt according to the province.

Given that 62.2% of all salt is non-iodized, a significant proportion of Cambodians likely do not have access to iodine through salt.

## 4. Discussion

The dramatic shift in the prevalence of non-iodized salt in Cambodia to 62%, as seen in all salt samples analyzed in 2014, is of major concern to the sustainability of the national iodization project. The substantial decrease in appropriately iodized salt on the Cambodian market (less than 1% of coarse salt and 23% of fine salt) shows how fragile the program is after four years of no premix support (free potassium iodate) from UNICEF or other development partners.

Our findings contradict internal monitoring from local producers. According to monthly SPCKK reports from 2008 to 2014 submitted to the Ministry of Planning (personal communication), more than 90% of 27,000 samples had a concentration between 20 and 60 mg/kg. After our study, we can be skeptical of the legitimacy of the internal control or on the stability of iodine. The lack of ongoing external quality control since the last national survey is of great concern and threatens the program’s sustainability. In 2004, M.B. Zimmermann [8] emphasized that no program will last if national governments, donors, consumers, and the salt industry do not make a long-term commitment.

The stability of the iodine itself in Cambodian salt could be questioned, but in a 1998 review from several global studies, the losses of iodine from salt stored for up to six months with solid low-density polyethylene packaging ranged from 10%–15% [9]. Therefore, unless the packaging or other factors are different to those observed in 2008 and 2014, it would be difficult to blame the stability of the iodization process. But we should not underestimate it as in several markets, branded iodized salts were sold loosely, which could affect the iodine content. Coarse salt is also often not as well packaged as fined salt, thereby affecting its stability. In 2008, an American study showed that loss of iodine over time with exposure to air at 22 °C could reach almost 50% at relative humidity of 65% and almost 90% at 80% [10]. Even if Potassium iodate (used in Cambodia) is more stable under adverse climatic conditions than Potassium iodide (used in the USA) [11], it is important not to underestimate the potential losses in Cambodia due to humidity (average humidity is already 70%–80% in Cambodia) and light exposure for future investigation.

Looking at the map of the proportion of fine salt marketed being non-iodized in different provinces of Cambodia, it is surprising to observe that salt producing provinces have also high level of non-iodized salt. According to Conkle *et al.* [4], all salt must be iodized before leaving Kep and Kampot provinces but unfortunately in these provinces approximately half of fine salt is not. This would be in line with the assumption made previously [4] that the low coverage is due to leakage on non-iodized salt from production areas. According to the international food fortification guidelines [12], at least 80% of individual samples need to meet the legal minimum level and less than 20% should be above, but never too far from the maximum tolerable level. If these criteria are not fulfilled, then a warning statement must be provided and more frequent inspection should be planned at the level of the producers and the market. Our study shows clearly that there is an emergency to act.

The high level of non-iodized salt questions the current iodization practices of several local manufacturers but also the impact of Vietnamese and Thai imports in Cambodia. The price variance between Cambodian domestic salt (USA $65 per metric tons) and Vietnamese salt (USA $30–$40 per metric tons) [4] encourages non-iodized Vietnamese salt producers to smuggle their product as no routine government monitoring is in place. In Vietnam, the 2005 revised decree on the production of iodized salt failed to uphold the mandatory iodization requirement [13]. Since then, Vietnamese producers have stopped iodizing their salt and are now exporting non-iodized in Cambodia as well.

It is even more important to act as it might have an impact on Cambodian health. The increase of non-iodized salt in the market will definitely influence the iodine status of the Cambodian population. In Vietnam, since 2005, the iodine concentration in urine among women of reproductive age (15–44 years of age) fell from 122 μg/L in 2006 to 83 μg/L in 2009 [14], which might be explained by the significant decrease of adequate iodized salt in the Vietnamese market. Similar findings were observed in several countries. For example in Guatemala, once the program faced challenges with less than 50% of the households accessing iodized salt, new cases of cretinism have appeared [8]. This was similar in former Soviet republics such as Azerbaijan [15], Kazakhstan and Kyrgyzstan [8]. In Cambodia, one unpublished study implemented in June 2014 on urinary iodine concentration from 2300 schoolchildren in Kampong Speu Province showed that the median declined to 167 μg/L from 236 μg/L seen in the 2008 and 2011 national studies [2,5]. Those surveys indicate clearly the importance of iodized salt to prevent and poor urinary iodine concentration.

The current standard (30–59.9 mg/kg) in Cambodia could be seen as a burden for the salt industry due to the cost implication and the lack of enforcement at the border against neighboring countries exports of non-iodized salt. The World Health Organization, UNICEF and the International Council for Control of Iodine Deficiency Disorders have recommended that iodized salt should provide 150 μg of iodine per person per day. If the program was appropriately implemented with an average iodization of 30 mg/kg, the consumption of 10–15 g of salt (actual Khmer consumption) would provide 300–450 μg of iodine per person per day. Therefore, the Cambodian Government could reduce this burden and pave the way for better compliance from the salt producers if they reduced their standards to 15–30 mg/kg, as is the case in many other countries [16] and reach the appropriate amount of iodine per day (150 μg). The future updated standard should be in line with neighboring countries such as Thailand and soon in Vietnam in order to ease trade given upcoming ASEAN free trade agreements.

But the enactment of mandatory legislation on its own is not sufficient as highlighted in this paper. Industry partners have argued that iodizing salt represents a competitive disadvantage as no enforcement is being implemented by the government against Vietnamese or Thai exports. It is therefore important to implement a well-designed monitoring system for quality control and assurance. Fortification standards have to become integrated in the industry licensing and registration processes. In order to sustain the iodization initiative, it is vital to ensure that every producer iodizes according to the standards. Rapid test kits are available and therefore should be more often use at the border to reassure the strong support of the government for a competitive and fair market.

### Limitation

The comparability of the two surveys could be questioned as the samples were not collected in the same way (household *vs.* market level). It is important to understand that those surveys are snapshots of salt iodization at the time of the survey. Even if those studies are using different methodologies for the collection of the salt: (I) through schoolchildren in 2008 and 2011; and (II) through the market in 2014, we can consider that they will provide similar information on the tendency of salt producers to follow or not the mandatory legislation on salt iodization. Markets are a reflection of consumer preference while schoolchildren are providing what is actually available at the households. Both should be aligned as supplies and demands are correlated. In 2014, to overcome the difference between methodologies, the provincial surveyors have collected salt from biggest markets of the 25 provinces chosen randomly and we consider that the salt sold represent the demand from consumers. It is also important to point out that according to recent unpublished data from the Ministry of Commerce presented to NSCIDD, the prevalence of non-iodized salt was similar and even worse than our findings with only 22% of the fine salt being iodized and only 4% of the coarse. Therefore, both analyses (the current paper and the ministry of commerce) show the urgency to enforce current legislation and comfort the authors on their findings.

## 5. Conclusions

Having strong iodized salt legislation alone does not lead to sustainability once donors have stopped their support. The findings of this study indicate that iodization of salt in Cambodia has decreased from being well implemented to marginally iodized. This reduction illustrates the lack of periodic monitoring and enforcement from government entities that could report on timely progress made or lost over the year. To ensure that iodine deficiency disorders do not remerge, Cambodia can learn from the experiences in other countries such as Switzerland, where, for instance, periodic surveillance and prudent adjustments to the iodine level in salt has maintained adequate iodine nutrition [17].

Therefore it is recommended to implement the following eight interventions: (I) test the stability of iodine within Cambodian salt and market settings; (II) assess the impact of low iodization on the Cambodian iodine status; (III) assess Cambodian salt producers’ bottleneck; (IV) develop a monitoring system with the food administration authority to ensure that domestic and imports are iodized; (V) ensure that appropriate national budget is allocated to the enforcement of the legislation; (VI) develop regional standards and regulation for iodized salt including penalties [18]; (VII) ensure that fortification standards including iodization are integrated in the annual industry licensing and registration and finally (VIII) sensitize the population on fake labeling by industry promoting fortification including iodization.

## Supplementary Files

Supplementary File 1
