# Supplementary material for: Iodized Salt in Cambodia: Trends from 2008 to 2014
Source: Nutrients. 2015 May 29;7(6):4189–98. doi: 10.3390/nu7064189 (PMC4488780; doi:10.3390/nu7064189)
Supplement: Supplementary File 1 [file nutrients-07-04189-s001.pdf]

Supplementary Information:

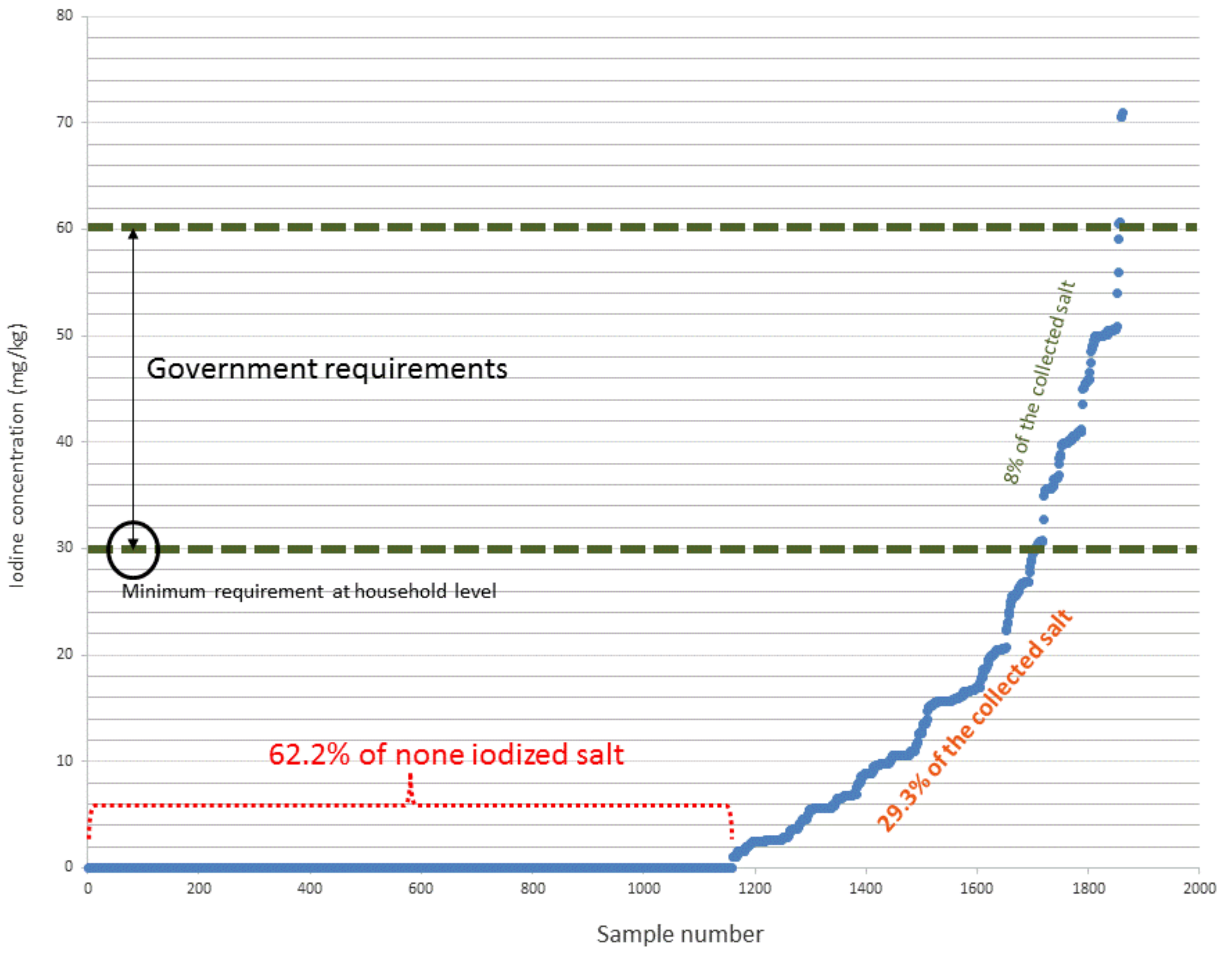

Supplementary Figure. 2014 Iodine values (mg/kg) in all salt (sorted by lowest to highest iodine value).
